# Correlation of MET and PD-L1 Expression in Malignant Melanoma

**DOI:** 10.3390/cancers12071847

**Published:** 2020-07-09

**Authors:** Kyu Young Song, Sabina Desar, Thomas Pengo, Ryan Shanley, Alessio Giubellino

**Affiliations:** 1 Department of Laboratory Medicine and Pathology, University of Minnesota, Minneapolis, MN 55455, USA; songx047@umn.edu (K.Y.S.); sdeshar1@gmail.com (S.D.); 2 Masonic Cancer Center, University of Minnesota, Minneapolis, MN 55455, USA; 3 University of Minnesota Informatics Institute, University of Minnesota, Minneapolis, MN 55455, USA; tpengo@umn.edu; 4 Masonic Cancer Center Biostatistics Core, University of Minnesota, Minneapolis, MN 55455, USA; shan0219@umn.edu

**Keywords:** MET, PD-L1, melanoma, metastasis

## Abstract

The proto-oncogene MET, the hepatocyte growth factor (HGF) receptor, is a transmembrane receptor tyrosine kinase (RTK) with a prominent role in tumor metastasis and resistance to anti-cancer therapies. Melanoma demonstrates relatively frequent MET aberrations, including MET gene amplification. Concurrently, programmed death-ligand 1 (PD-L1), with its ability to evade anti-tumor immune responses, has emerged as a prominent therapeutic target in melanoma and other malignancies and its expression is used as a predictive biomarker of response to immunotherapy. We performed immunohistochemistry analysis of MET and PD-L1 in 18 human melanoma cell lines derived from both primary and metastatic lesions, and in a human melanoma tissue microarray containing one hundreds melanocytic lesions, including primary cutaneous melanomas, primary mucosal melanomas, metastatic melanomas and benign melanocytic nevi as controls. After color deconvolution, each core was segmented to isolate staining and calculate the percentage of positive cells. Overall, MET expression was higher in tumors with increased PD-L1 expression. Moreover, a robust correlation between MET and PD-L1 expression was found in samples from metastatic melanoma and not in primary cutaneous or mucosal melanoma. These data suggest that relative expression levels of these proteins in combination is a marker of advanced disease and testing for expression of these markers should be considered in patients with melanoma.

## 1. Introduction

Melanoma is the leading cause of death in patients with cutaneous malignancies and its incidence has been rapidly rising over the past 30 years. According to the American Cancer Society, 100,350 estimated new cases of melanoma and 6850 estimated deaths related to melanoma will occur in 2020 [1]. While the five-year survival rate is up to 98% if melanoma is diagnosed at an early stage, this rate drops significantly if the disease is diagnosed at a late stage and has metastasized to distant organs [2]. While a better understanding of the molecular basis of this cancer and its microenvironment has resulted in relatively successful novel therapeutic options, resistance ensues and there is still need for a better way to select for patients who will more likely benefit from current therapies and to explore novel combination strategies.

The proto-oncogene MET, the hepatocyte growth factor (HGF) receptor, is a transmembrane receptor tyrosine kinase (RTK) with a prominent role in tumor metastasis and resistance to anti-cancer therapies [3]. Dysregulation of the HGF/MET signaling pathway has been demonstrated in a wide range of malignancies, including malignant melanoma [4,5]. A recent large whole genome sequencing (WGS) analysis of melanomas has demonstrated relatively frequent MET aberrations, including MET gene amplification, single nucleotide variations/deletions, and structural variants [6].

The pleiotropic effect of the HGF/MET signaling pathway include also a role in modulation of the immune response, including involvement in dendritic cell function [7] and neutrophilic antitumoral response [8]. This function has been postulated to be involved in the potential acquisition of resistance to immunotherapy treatments, such as monoclonal antibodies targeting PD-1 [7].

Programmed death-ligand 1 (PD-L1) is a transmembrane protein encoded by the CD274 gene, located on chromosome 9, and is expressed by antigen presenting cells (APCs) and tumor cells [9]. The PD-1/PD-L1 axis, with its ability to evade the anti-tumor immune response, has emerged as a prominent therapeutic target in melanoma and other malignancies [10] and expression of PD-L1 is used as a predictive biomarker of response to immunotherapy [11]. PD-L1 expression can be induced either by cytokines (INF-gamma) or by activation of an oncogene. For example, mutations in receptor tyrosine kinase pathways, such as epidermal growth factor receptor (EGFR), have been shown to induce PD-L1 expression in lung tumors [12] and this overexpression in cancer cells can block anti-tumor immunity, resulting in immune escape [13], which can be overcome by PD-1/PD-L1 inhibition by restoring tumor-specific T-cell immunity.

An interplay between MET and PD-L1 has been demonstrated in several malignancies. For example, MET expression has been shown to promote upregulation of PD-L1 in renal cancer cells [14] and expression of PD-L1 and PD-L2 are upregulated in MET-amplified gastric and lung tumor cells [15]. However, the relationship between MET and PD-L1 expression in human malignant melanoma is not well characterized.

In the present study, we surveyed by immunohistochemistry MET and PD-L1 expression in melanoma cell lines and in a human tissue microarray of cutaneous melanomas, mucosal melanomas and metastatic melanomas, with the goal of comparing their expression pattern and to explore a possible correlation between expression of these two proteins involved in tumor progression and immune evasion.

## 2. Results

### 2.1. Expression of MET and PD-L1 in Melanoma Cell Lines

**Figure 1:**
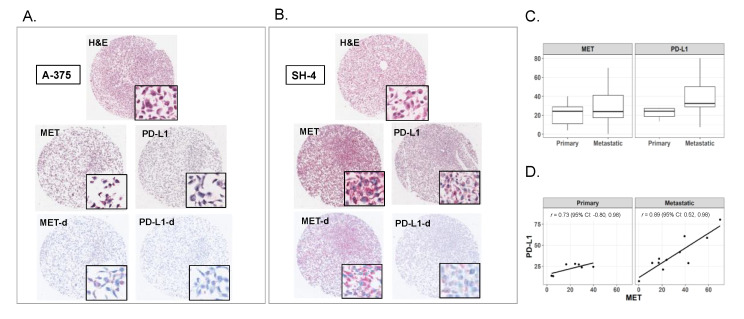
Histopathology images of MET and programmed death-ligand 1 (PD-L1) expression levels in a representative primary melanoma (A-375, panel **A**) and a metastatic melanoma (SH-4, panel **B**) cell lines. Hematoxylin and eosin (H&E), MET, and PD-L1 immunostained slides from the cell microarray (CMA) are represented (full cores, 4×; insets, 40×), as well as color-deconvolved images for MET (MET-d) and PD-L1 (PD-L1-d). Boxplot with the box representing the interquartile range and a dark line representing median MET and PD-L1 in seven primary melanomas and 11 metastatic melanomas (**C**). Correlation of MET and PD-L1 in primary and metastatic cell lines, with Pearson’s r values and 95% confidence interval (**D**).

We analyzed the expression of MET and PD-L1 in 18 human melanoma cell lines including seven primary and 11 metastatic cell lines. Immunohistochemical staining for MET and PD-L1 revealed a wide range of expressions in these cell lines. Representative histopathologic images of a primary melanoma cell line (A-375) and a metastatic melanoma cell line (SH-4) are shown in Figure 1 (A and B, respectively). Overall, expression of PD-L1 was higher in metastatic melanoma cell lines compared to primary melanoma cell lines (Figure 1C). Median PD-L1 was 32 (interquartile range: 29–50) for metastatic and 24 (19–27) for primary cell lines (Wilcoxon rank sum test *p* = 0.02), and the highest value was in a metastatic cell line. Expression of MET was similar in primary and metastatic cell lines, although the interquartile range was higher for metastatic (17–41) than primary (11–29) tumor cells (Wilcoxon rank sum test *p* = 0.44), and the highest value of MET was in a metastatic cell line (Table S1).

MET and PD-L1 values were correlated among primary melanoma cell lines, with Pearson’s *r* = 0.73, with a notable wide confidence interval. For metastatic cell lines, the Pearson’s correlation score was higher, with an *r* = 0.89 (Figure 1D).

### 2.2. Expression of MET and PD-L1 in a Human Melanoma Tissue Microarray

**Figure 2:**
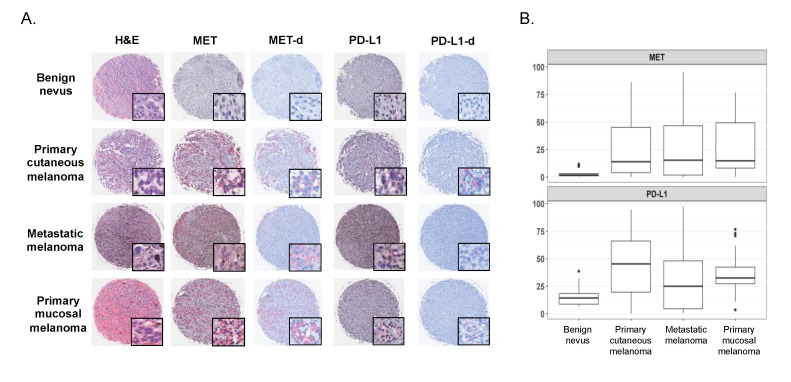
MET and PD-L1 expression in a representative benign nevus, primary cutaneous melanoma, metastatic melanoma, and primary mucosal melanoma. Hematoxylin and eosin (H&E), MET, and PD-L1 immunostained slides from the tissue microarray (TMA) are represented (full cores, 4×; insets, 40×), as well as color-deconvolved images for MET (MET-d) and PD-L1 (PD-L1-d) (panel **A**). Boxplot with the box representing the interquartile range and a dark line representing median MET and PD-L1 in benign nevi, primary cutaneous melanoma, metastatic melanoma, and primary mucosal melanoma (panel **B**).

One-hundred melanocytic lesions were evaluated for expression of MET and PD-L1 in a human tissue microarray (TMA) with benign nevi (17 patients), primary cutaneous melanomas (42 patients), primary mucosal melanomas (20 patients), and metastatic melanomas (21 patients). Representative images for each histologic subtype are represented in Figure 2A.

As in the cell lines, MET and PDL1 expression varied widely across the lesions in the TMA specimens, and the lowest levels of both MET and PD-L1 expression were detected in benign nevi, as expected. Primary cutaneous melanoma, primary mucosal melanoma and metastatic melanomas showed comparable levels of MET (Figure 2B). Median MET was 2 (interquartile range: 1, 3) for benign nevi, 14 (4–45) for primary cutaneous melanoma, 15 (2–47) for metastatic melanoma, and 15 (8–49) for primary mucosal melanoma (Table S2). Interestingly, no nevi had MET expression above a threshold of 20% positive cells and only one was above a threshold of 10%. MET expression by more than 20% of cells was present in 40% (17 of 42) of primary cutaneous melanoma, 45% (nine of 20) of primary mucosal melanoma, and 33% (seven of 21) of metastatic melanoma.

When measuring PD-L1, the median was 16 (9–18) for benign nevi, 45 (20–66) for primary cutaneous melanoma, 25 (5–48) for metastatic melanoma, and 33 (27–42) for primary mucosal melanoma (Table S2). Using the same threshold as described for MET, 12% (2 of 17) of nevi, 70% (30 of 42) of primary cutaneous melanoma, 80% (16 of 20) of primary mucosal melanoma, and 50% (11 of 21) of metastatic melanomas demonstrated PD-L1 expression by more than 20% of cells.

**Figure 3:**
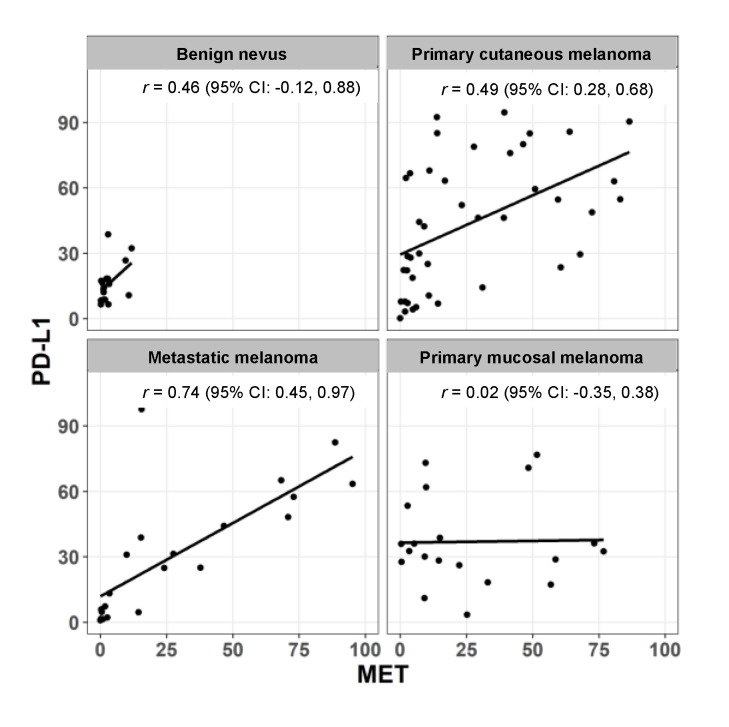
Correlation of MET and PD-L1 in benign nevi, primary cutaneous melanoma, metastatic melanoma, and primary mucosal melanoma, with Pearson’s r values and 95% confidence interval.

We then calculated the correlation between MET and PDL1 expression in each category of melanocytic lesions. As shown in Figure 3, there was a modest correlation for benign nevi and primary cutaneous melanoma, with Pearson’s correlation coefficients of 0.46 and 0.49, respectively, and no correlation in primary mucosal melanoma (*r* = −0.02). In contrast, MET and PD-L1 expressions were highly correlated in metastatic melanoma (*r* = 0.74).

## 3. Discussion

Metastatic malignant melanoma remains a major cause of death among patients with cutaneous malignancies, despite the introduction of novel therapeutic approaches such as immunotherapy. The PD-1/PD-L1 axis [16] have emerged as a major and effective immune checkpoint target for immunotherapy. Resistance to drugs targeting this axis eventually emerges [17] through several mechanisms, some of which have been characterized. For example, activation of canonic oncogenic signaling pathways, such as those driven by receptor tyrosine kinases, are now recognized to play an important role in tumor resistance to this therapy. Thus, a viable strategy to overcome resistance is to combine immunotherapy with conventional targeted therapies, such as inhibitors of receptor tyrosine kinases [18]. An important role in the selection of patients who will benefit from these therapies is played by accurate assessment of expression of these targets in tumor tissue sections. Indeed, in the era of personalized medicine, the prediction of patient’s drug response has become an important prerequisite for therapeutic intervention [19].

In our study, we analyzed the expression profile of a receptor tyrosine kinase, MET, and the checkpoint protein PD-L1 in tumor cells. MET activation in tumor cells mediates a variety of cellular functions, including cell proliferation, metastasis, and angiogenesis [5]. In addition, several lines of evidence point out a prominent role of the MET/HGF axis in tumor progression and resistance to therapy of several malignancies, including malignant melanoma [20]. For example, in a case of acral melanoma with KIT mutation, targeting MET with a selective inhibitor successfully overcame resistance to KIT inhibition, as confirmed also in cell line studies [21]. Another study has established that MAPK pathway inhibition following BRAF inhibitor treatment induced rapid increases in MET and GAB1 expression [22] and MET amplification was also observed to co-exist with BRAF hotspot mutations and represent the most common amplification among RTKs in melanomas [6]. Moreover, downstream effects of MET phosphorylation include activation of the MAPK signaling pathway, where more than 80% of melanomas harbors alterations [23].

It is interesting to notice that MET also appears to have a role in the regulation of immunity, as demonstrated by the key role of its ligand, HGF, in the regulation of autoimmunity and inflammation [24]. More recently a subpopulation of CD8 positive cytotoxic T-cell has been found to express MET, although at low levels, further linking its pathway to a role in tumor immunity [25]. Moreover, HGF has been linked to increased expression of PD-L1 in dendritic cells and CTLA-4 in T-cells, with a role in the induction of immune tolerance [7,26], further linking MET signaling to immune checkpoint pathways.

Our cell line data show that expression of MET and PD-L1 lies in a broad range of expression values, highlighting the wide variability particularly in metastatic cell lines, as shown in the broad range and interquartile data. While MET expression median values were similar in primary and metastatic melanoma cell lines, we found a trend in higher values in the metastatic cell lines, which comprised the cell line with the highest expression value. At the same time, PD-L1 showed a clear statistical significant increase in metastatic cell lines when compared to primary melanoma cell lines. Most importantly, when looking at the correlation between expression of the two proteins, we identified a higher correlation in metastatic cell lines versus primary cell lines.

In tumor tissue samples from the TMA, all melanoma subtypes demonstrated statistically significant higher values for both MET and PD-L1 when compared with benign nevi. These data are in concordance with similar analysis in other types of malignancies. It was interesting to observed a large degree of variability in expression of both proteins in primary cutaneous and primary mucosal melanoma, as well as in metastatic melanoma, as shown by the broad range of interquartile values and the variability outside the upper and lower quartiles. While there is an apparent trend in higher PD-L1 values in the primary melanoma samples compared with the metastatic melanomas, the difference is not statistically significant, as the p values of this comparison is 0.11, with wide overlapping interquartile values. It is also important to notice that a lower number of metastatic melanomas (21) were present in the TMA when compared with the number of primary cutaneous melanoma (42).

When we correlated the expression of both proteins, benign nevi and primary cutaneous melanoma had a relatively poor correlation, while no correlation was seen in primary mucosal melanoma. In contrast, metastatic melanoma showed a strong correlation between MET and PD-L1 expression. Of note, when comparing the correlation of MET and PD-L1 in cell lines CMA compared with patient tissue sample from the TMA, while metastatic cell lines showed an higher correlation between the two markers compared to the primary melanoma cell line, the difference in r values was minimal compared with the difference in the same comparison in the TMA. A possible interpretation of this phenomenon likely lies in the intrinsic uniformity of immortalized cell lines when compared with actual tumor samples.

In our study we took advantage of quantitative digital analysis of high resolution images and we used a recently described open source software (QuPath) for digital pathology and image analysis [27,28]. The advantages of this methodology includes color deconvolution and segmentation in a stepwise protocol that can be adopted across different samples and different experiments, maintaining reproducibility across studies. It also allows for a sensitive assessment of each individual staining and is able to detect low levels of signal that may be otherwise below the limit of detection in a qualitative or semiquantitative assessment.

Moreover, compared with a traditional categorical and qualitative assessment, our method allows the measurement of numerical values on a continuous scale, improving accuracy of chromogenic immunohistochemistry analysis, and allowing increased sensitivity and a quantitative assessment. It is also an improvement to the semiquantitative assessment of the percentage of positive cells that may also carry interobserver variability.

There are some limitations to the current study. One limitation is the relatively small sample size for both the CMA and the TMA. Expanding the cohort of patients with melanoma, in retrospective or prospective studies, will help to confirm our findings. Moreover, regarding the TMA, unfortunately we had limited clinical information and no knowledge about outcomes or therapeutic regimens in these patients, thus, limiting the possibility to further explore these proteins as prognostic and predictive markers in our assessment. Thus, future studies with a larger cohort and well annotated samples will help to answer those questions and support the rationale of using MET and PD-L1 in combination as predictive and prognostic markers in malignant melanoma. The recent success in the treatment of melanoma demonstrates that mechanism-based strategies of molecular markers and therapeutic targets that are overexpressed or otherwise dysregulated in tumors are effective. This is exemplified by the assessment, in melanoma tissue samples, of the expression of BRAF mutations, which is currently the most robust predictive biomarker influencing eligibility to targeted therapy with vemurafenib and dabrafenib [29].

The use of single agent therapy has revealed to be limited as resistance to these regimes develop [30,31]. This points out the need for combined therapy to reach durable and effective response. This has been demonstrated by the success of combination immunotherapies [32] and the use of combined BRAF and MEK targeted inhibition [33]. A combination of immune checkpoint inhibitors with targeted therapies is the next logical step that is currently being explored in ongoing clinical trials.

Our data on the strong correlation between MET and PD-L1 in metastatic lesions of melanoma, support the idea that these markers follow a proportional pattern of expression in advanced lesions. There is sporadic evidence from the literature which points, separately, to both MET and PD-L1 as potential biomarker of response to therapy selectively targeting these proteins in several tumor settings. For MET, for example, in a phase I clinical trial testing the humanized anti-MET antibody Onartuzumab, patients with metastatic, chemotherapy-resistant gastric carcinomas, and high MET expression (defined qualitatively as > 50% of tumor cells with 2+ and 3+ staining) showed complete response with improved progression free survival (PFS) and overall survival (OS) [34]. Additionally, in a phase II study using the same anti-MET therapeutic antibody (in combination with erlotinib) in patient with advance non-small cell lung cancer, MET expression, as accessed by immunohistochemistry, represented a robust predictor of OS and PFS [35]. There is also further evidence that MET expression can identify patients who will develop resistance to current melanoma treatments. For example, Hugo et al. [31] have shown that a subset of melanomas with acquired resistance to MAPK inhibitors overexpress MET. Additional evidence from the literature point out to MET expression as a proximal biomarker for response to MET-selective inhibitors [36,37].

We also have evidence from the literature that PD-L1 expression, although an imperfect marker, can predict the likelihood of response to anti-PD1/PD-L1 axis therapy. In absence of better biomarkers, at the request of treating oncologists, pathologists frequently measure PD-L1 expression by immunohistochemistry to help guide the use of immunotherapy. While selecting patients that will benefit from immunotherapies remains a challenge, PD-L1 expression by IHC still hold some value, as tumor overexpressing PD-L1 frequently demonstrate to have improved outcomes to anti-PD1 therapies; however, the response to this therapy in patients with low PD-L1 levels demonstrate that it may not be an exclusive predictive biomarker to select patients for these therapeutic regimens [38]. This further emphasizes that exploration of other markers, alone or in combination, is needed.

Further studies, using functional assays, will help to determine whether this correlation can be used to select patients for therapeutic interventions and whether selective inhibitors targeting these proteins and their pathways in combination will be effective in patient with advanced melanoma.

## 4. Materials and Methods

### 4.1. Cell Culture and Cell Microarray

Human cell lines (A2058, A-375, G-361, RPMI-7951, SH-4, SK-MEL-1, SK-MEL-3, SK-MEL-24, and SK-MEL-28) were obtained directly from ATCC and were cultured following ATCC’s recommendations. The WM35, WM115, WM164, WM278, WM793, WM852, WM1341D, 451Lu, and 1205Lu cell lines were obtained from the Wistar Institute and cultured with tumor specialized media containing 2% FBS. The characteristics of these melanoma cell lines are highlighted in Table S3.

To prepare cell pellet blocks, cells were grown in T-75 flasks to near confluence then washed three times with PBS and fixed with 10% neutral-buffered formalin (NBF) in flasks overnight. After fixation, cells were gently scraped and transferred to a conical tube and centrifuged to ~300× *g* for 5 min at room temperature (RT). Formalin supernatant was then removed and pellets were washed with PBS. Finally, cell pellets were re-suspended in 80% ethanol and paraffin embedded in cell blocks. A tissue microarray (TMA), with 1.5 cm cores for each cell line, was then derived from the cell blocks. Two TMA slides were stained for MET and two were stained for PD-L1. The average percentages of positively stained cells and FastRed mean intensity were calculated.

### 4.2. Tissue Microarray and Patient Characteristics

TMA slides with 100 cores were purchased from Biomax (Cat. #ME1004 g; US Biomax, Inc., Derwood, MD, USA). Available clinicopathological characteristics are summarized in Table S4. The mean age of the patients from this TMA was 50 years (range 0.5–84 years). The study included 58% (58) males and 42% (42) females. Staging (TNM and clinical staging) was only provided for 48 patients, which include 42 cases of primary cutaneous melanoma and six cases of primary mucosal melanoma. Overall, the TMA included 42 cases with primary cutaneous melanoma, 20 primary mucosal melanomas (including malignant melanoma from vulva, rectum, stomach and esophagus), 21 cases were obtained from metastatic sites including lymph nodes, and 17 cases were benign melanocytic nevi.

Two TMA slides were stained for MET and two were stained for PD-L1 (see representative images in Figure S1). The average percentage of positively stained cells and FastRed mean intensity were calculated.

### 4.3. Immunohistochemistry (IHC)

Unstained TMA sections (4 µm) were de-paraffinized and rehydrated using standard methods. For antigen retrieval, slides were incubated in 6.0 pH buffer (Reveal Decloaking reagent, Biocare Medical, Concord, CA, USA) in a steamer for 30 min at 95–98 °C, followed by a 20 min cooldown period. Subsequent steps were automated using an immunohistochemical staining platform (Intellipath, Biocare, Pacheco, CA, USA). Endogenous peroxidase activity was quenched by slide immersion in 3% hydrogen peroxide solution (Peroxidazed, Biocare) for 10 min followed by TBST (Tris-Buffered Saline Tween) rinse. A serum-free blocking solution (Background Sniper, Biocare Medical, Concord, CA, USA) was placed on sections for 10 min. Blocking solution was removed and slides were incubated in primary antibody diluted in 10% blocking solution/90% TBST for 60 min at room temperature. Rabbit monoclonal anti-MET (clone D1C2 XP(R) (Cell Signaling, Denver, MA, USA;1:50) was followed by a TBST rinse and detection with Novocastra Novolink Polymer Kit (Leica Microsystems Inc., Buffalo Grove, IL, USA) using the manufacturer’s specifications. Slides then proceeded with TBST rinse and detection with diaminobenzidine (DAB) (Covance, Dedham, MA, USA). Slides were incubated for 5 min followed by TBS rinse then counterstained with CAT Hematoxylin (Biocare, Concord, CA, USA) for 5 min. Finally, slides were dehydrated and coverslipped.

Rabbit monoclonal anti-PD-L1 (clone 28-8, Cell Marque, Rocklin, CA, USA, 1:200) was followed by a TBST rinse and biotinylated anti-rabbit (Vector Labs, Burlingame, CA, USA, 1:200) was applied for 30 min. The slides were again rinsed with TBST and 4+ Streptavidin-Alkaline Phosphatase label (4 + SA-AP) (Biocare Medical, Concord, CA, USA, RTU) was applied for 30 min at room temperature. Slides proceeded to a TBST rinse and detection with WARP Red Chromagen (Biocare Medical, Concord, CA, USA) according to manufacturers’ specifications. Following detection, slides were rinsed well in running tap water and counterstained for 1 min in CAT Hematoxylin (1:2) (Biocare Medical, Concord, CA, USA). Slides were then rinsed in tap water and placed for 2 min in PureView PH Blue (Cancer Diagnostics, Durham, NC, USA), followed by 5 min tap water rinse; then slides were air dried. When completely dry, slides were dipped in xylene and coverslipped with Permount mounting medium (Fisher, Fair Lawn, NJ, USA).

### 4.4. Image Analysis

TMA slides were scanned using an Aperio scanner, with a 40× objective. The high-resolution images were analyzed using QuPath [27] version 0.1.2. The workflow consisted of (i) color deconvolution, (ii) identifying the CMA or TMA cores, (iii) segmenting the tissue region in each core, (iv) isolating nuclear and cytoplasmic regions of interest, (v) estimating the abundance of the deconvolved red component in each cell, and finally (vi) calculating the percentage of positive cells in each core. Three-color deconvolution was performed using vectors calibrated visually on the image data, following the procedure outlined in the software documentation. All images were deconvolved using the same stain vectors. Steps (iii) to (v) were performed using the default algorithms in QuPath. For step (vi), each cell was considered positive if the red component intensity was above a threshold, calculated independently for each CMA or TMA slide as the average between the 5th and 95th percentile of red intensities across the slide. All algorithms and parameters for the analysis in QuPath were recorded in a script for repeatability. Color composition of blue and red are components of the color-deconvolved image. The original image was deconvolved using the “Color Deconvolution” plugin in Fiji (download data May 5th 2020) with the “FastRed FastBlue DAB” color settings, producing a blue, a red, and a brown image. The blue and red images were converted to RGB and merged using the “Image Calculator” with “Min” settings. No contrast adjustments were made.

### 4.5. Statistical Analysis

Statistical analysis was primarily descriptive. Correlation between percentage of MET—and PD—L1-positive cells was calculated using Pearson’s correlation coefficient (*r*) and its 95% confidence interval calculated by the 2.5 and 97.5 percentiles of 500 bootstrap resamplings.

## 5. Conclusions

Our data show that a robust correlation exists in metastatic lesions and not in primary lesions or in mucosal melanoma, and suggest that relative levels of expression of these proteins in combination is a marker of advanced disease. These data, while preliminary, suggest that testing by immunohistochemistry for expression of these markers should be considered in patients with melanoma. We also defined a quantitative method, which is sensitive and allow unbiased comparison across samples and experiments and will help to guide assessment of these two proteins in future studies. We hope that our findings will inform and stimulate further mechanistic and functional studies as a bridge to consider those two proteins as biomarker and targets for therapy for patients with melanoma.

## Supplementary Materials

The following are available online at https://www.mdpi.com/2072-6694/12/7/1847/s1, Figure S1. Tissue microarray images with marked position by rows and columns coordinates (see corresponding cases detailed in Table S4): hematoxylin and eosin (H&E), MET, and PD-L1 immunostained slides, Table S1. Median (with interquartile range) and range values for primary melanoma cell lines and metastatic melanoma cell lines, Table S2. Mean (with 95% confidence interval) and median (with interquartile range) for benign nevi, cutaneous melanoma, metastatic melanoma, and mucosal melanoma in the TMA, Table S3. Melanoma cell lines evaluated in the cell line microarray (CMA), Table S4. Demographic and available clinical information for the TMA samples.
