# Supplementary material for: Correlation of MET and PD-L1 Expression in Malignant Melanoma
Source: Cancers (Basel). 2020 Jul 9;12(7):1847. doi: 10.3390/cancers12071847 (PMC7408820; doi:10.3390/cancers12071847)
Supplement: Supplementary file 1 [file cancers-12-01847-s001.zip › cancers-829177-layout-supplementary/cancers-829177-layout-supplementary.docx]

Supplementary Materials

Correlation of MET and PD-L1 Expression in Malignant Melanoma

Kyu Young Song, Sabina Desar, Thomas Pengo, Ryan Shanley and Alessio Giubellino


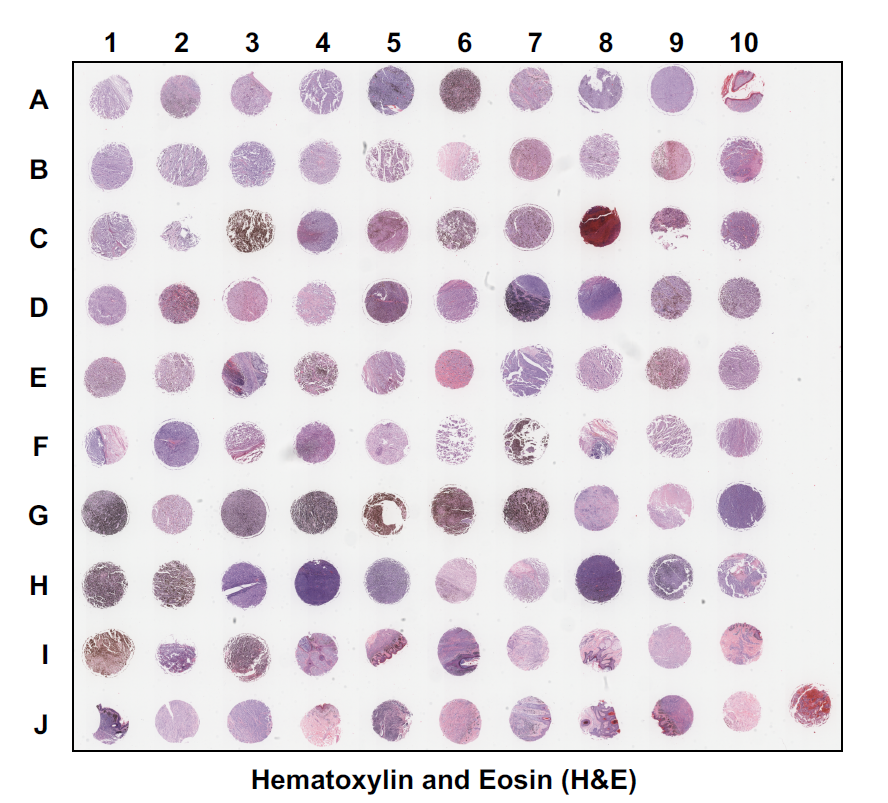


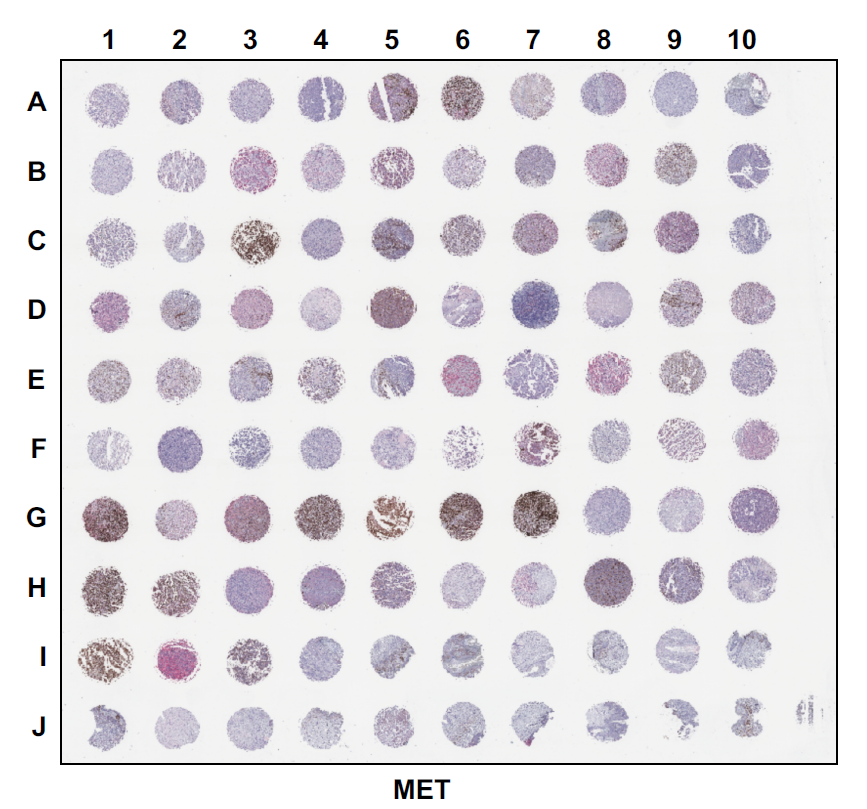


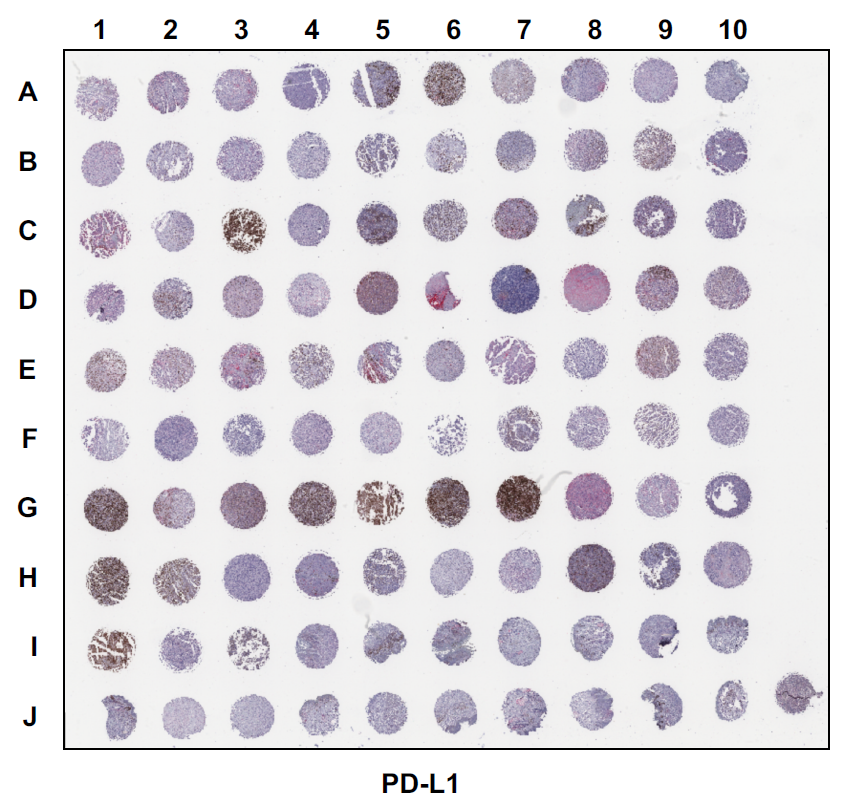


**Figure S1.** Tissue microarray images with marked position by rows and columns coordinates (see corresponding cases detailed in Table S4): hematoxylin and eosin (H&E), MET, and PD-L1 immunostained slides.

**Table S1.** Median (with interquartile range) and range values for primary melanoma cell lines and metastatic melanoma cell lines.

|  | Primary melanoma  (*N* = 7) | Metastatic melanoma (*N* = 11) | *p* Value |
| --- | --- | --- | --- |
| MET |  |  | 0.441 |
| Median (Q1, Q3) | 24 (10, 28) | 24 (17, 41) |  |
| Range | 4–40 | 0.2–70 |  |
| PD-L1 |  |  | 0.016 |
| Median (Q1, Q3) | 24 (18, 27) | 32 (28, 50) |  |
| Range | 13–27 | 7–80 |  |

**Table S2.** Mean (with 95% confidence interval) and median (with interquartile range) for benign nevi, cutaneous melanoma, metastatic melanoma, and mucosal melanoma in the TMA.

|  | Benign nevi  (*N* = 17) | Primary cutaneous melanoma (*N* = 42) | Metastatic Melanoma  (*N* = 21) | Primary mucosal Melanoma  (*N* = 20) | *p* Value |
| --- | --- | --- | --- | --- | --- |
| MET |  |  |  |  | < 0.001 |
| Mean | 3 | 26 | 28 | 26 |  |
| 95% CI for Mean | 2,5 | 18, 34 | 15, 41 | 17, 36 |  |
| Median (Q1, Q3) | 2 (1, 3) | 14 (4, 45) | 15 (2, 47) | 15 (8, 49) |  |
| PD-L1 |  |  |  |  | 0.006 |
| Mean | 16 | 44 | 31 | 37 |  |
| 95% CI for Mean | 13, 20 | 34, 53 | 19, 43 | 32, 49 |  |
| Median (Q1, Q3) | 14 (9, 18) | 45 (20, 66) | 25 (5, 48) | 33 (27, 42) |  |

**Table S3,4.** please view at the excel file.

| 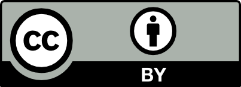 | © 2020 by the authors. Licensee MDPI, Basel, Switzerland. This article is an open access article distributed under the terms and conditions of the Creative Commons Attribution (CC BY) license (http://creativecommons.org/licenses/by/4.0/). |
| --- | --- |
